# Increased DNA methylation of *SLFN12* in CD4^+^ and CD8^+^ T cells from multiple sclerosis patients

**DOI:** 10.1371/journal.pone.0206511

**Published:** 2018-10-31

**Authors:** Brooke Rhead, Ina S. Brorson, Tone Berge, Cameron Adams, Hong Quach, Stine Marit Moen, Pål Berg-Hansen, Elisabeth Gulowsen Celius, Dipen P. Sangurdekar, Paola G. Bronson, Rodney A. Lea, Sean Burnard, Vicki E. Maltby, Rodney J. Scott, Jeannette Lechner-Scott, Hanne F. Harbo, Steffan D. Bos, Lisa F. Barcellos

**Affiliations:** 1 Computational Biology Graduate Group, University of California, Berkeley, Berkeley, CA, United States of America; 2 Genetic Epidemiology and Genomics Laboratory, Division of Epidemiology, School of Public Health, University of California, Berkeley, Berkeley, CA, United States of America; 3 Institute of Clinical Medicine, University of Oslo, Oslo, Norway; 4 Department of Neurology, Oslo University Hospital, Oslo, Norway; 5 Department for Mechanical, Electronics and Chemical Engineering, Oslo Metropolitan University, Oslo, Norway; 6 MS-Centre Hakadal, Hakadal, Norway; 7 Institute of Health and Society, Faculty of Medicine, University of Oslo, Oslo, Norway; 8 Translational & Integrative Analytics, Biogen, Inc., Cambridge, MA, United States of America; 9 Statistical Genetics & Genetic Epidemiology, Biogen, Inc., Cambridge, MA, United States of America; 10 Institute of Health and Biomedical Innovation, Queensland University of Technology, Brisbane, Australia; 11 Centre for Information Based Medicine, Hunter Medical Research Institute, Newcastle, Australia; 12 School of Biomedical Sciences and Pharmacy, University of Newcastle, Newcastle, Australia; 13 School of Medicine and Public Health, University of Newcastle, Newcastle, Australia; 14 Molecular Genetics, Pathology North, John Hunter Hospital, Newcastle, Australia; 15 Department of Neurology, John Hunter Hospital, Newcastle, Australia; Northwestern University Feinberg School of Medicine, UNITED STATES

## Abstract

DNA methylation is an epigenetic mark that is influenced by environmental factors and is associated with changes to gene expression and phenotypes. It may link environmental exposures to disease etiology or indicate important gene pathways involved in disease pathogenesis. We identified genomic regions that are differentially methylated in T cells of patients with relapsing remitting multiple sclerosis (MS) compared to healthy controls. DNA methylation was assessed at 450,000 genomic sites in CD4^+^ and CD8^+^ T cells purified from peripheral blood of 94 women with MS and 94 healthy women, and differentially methylated regions were identified using *bumphunter*. Differential DNA methylation was observed near four loci: *MOG*/*ZFP57*, *HLA-DRB1*, *NINJ2/LOC100049716*, and *SLFN12*. Increased methylation of the first exon of the *SLFN12* gene was observed in both T cell subtypes and remained present after restricting analyses to samples from patients who had never been on treatment or had been off treatment for more than 2.5 years. Genes near the regions of differential methylation in T cells were assessed for differential expression in whole blood samples from a separate population of 1,329 women with MS and 97 healthy women. Gene expression of *HLA-DRB1*, *NINJ2*, and *SLFN12* was observed to be decreased in whole blood in MS patients compared to controls. We conclude that T cells from MS patients display regions of differential DNA methylation compared to controls, and corresponding gene expression differences are observed in whole blood. Two of the genes that showed both methylation and expression differences, *NINJ2* and *SLFN12*, have not previously been implicated in MS. *SLFN12* is a particularly compelling target of further research, as this gene is known to be down-regulated during T cell activation and up-regulated by type I interferons (IFNs), which are used to treat MS.

## Introduction

Multiple sclerosis (MS) is a chronic inflammatory disease of the central nervous system, with onset during early adulthood, leading to demyelination and axonal degeneration that often progresses to physical and cognitive disability. The cause of MS is unknown, however, genetic and environmental factors, and interactions between them, are known to contribute to disease risk.[1–3] Variation in human leukocyte antigen (HLA) genes represent the strongest genetic susceptibility factor for MS, with the strongest signal in *HLA-DRB1*. In recent years, genome-wide association studies (GWAS) and custom chip-based studies have identified 200 MS-associated non-HLA loci.[4–6] Each of these genetic associations exerts only a modest effect size, and no genetic variant by itself is sufficient to cause MS, making the genetic contribution to MS etiology highly complex. The local linkage disequilibrium (LD) structure of most MS-associated loci makes the identification of true causal variants difficult. However, when inferring the most likely affected genes, a strong overrepresentation of immunologically relevant genes is observed, in particular for genes known to regulate T cell mediated immunity.[4,6]

MS heritability is not yet fully explained through the associated genetic variants, indicating that additional factors, such as epigenetic mechanisms, contribute to MS etiology. The term epigenetics describes heritable changes in gene regulation that do not alter the DNA sequence. DNA methylation, a widely studied epigenetic mechanism, is the addition of a methyl group to the fifth carbon position of cytosine at CpG dinucleotides. DNA methylation in gene promoter regions typically prevents transcription factors from binding and thereby silences gene expression, although other regulatory effects of DNA methylation are known.[7] While DNA methylation patterns can be inherited, they are also affected by environmental exposures such as tobacco smoke, diet, exercise, stress, and medications. Thus, DNA methylation may link environmental exposures and genetic variations to MS disease risk. DNA methylation associations have been shown in cancers,[8] and more recently, immune-mediated and neurodegenerative diseases,[9–11] including MS.[12–20]

Here, we investigate DNA methylation in CD4^+^ and CD8^+^ T cells purified from blood in Norwegian and Australian MS patients compared to healthy controls. Methylation differences in these cell types between MS patients and controls have been previously studied in both smaller cohorts.[13,15,16,20] More samples have since been added, and the Australian and Norwegian datasets have been combined to maximize statistical power. The current analysis represents the largest study to date on the role of DNA methylation of immune cells in MS. Epigenome-wide association analysis was performed to identify differentially methylated positions (DMPs) and differentially methylated regions (DMRs). Gene expression changes in whole blood corresponding to DMRs was used to validate our methylation findings in an independent dataset of MS cases and healthy controls.

## Results

Participant characteristics and proportion of cell type samples available for analyses are summarized in Table 1. CD4^+^ and CD8^+^ T cells were analyzed separately, and different subsets of cases were considered to evaluate potential bias based on treatment. In total, five sub-analyses were conducted: a) CD4^+^ T cells of all cases regardless of treatment vs. all controls; b) CD8^+^ T cells of all cases regardless of treatment vs. all controls; c) CD4^+^ T cells of cases not on treatment at the time of inclusion vs. all controls; d) CD4^+^ T cells of treatment-naïve cases vs. all controls; and e) CD8^+^ T cells of treatment-naïve cases vs. all controls (Table 2). There were insufficient CD8^+^ T cell samples to analyze cases off-treatment at time of inclusion. About 65,000 CpGs were removed from each dataset in quality control steps. The genomic inflation factor was close to one for all analysis strata, indicating that results were not considerably confounded after including surrogate variables (SVs) in the regression models. We compared estimated SVs to the measured variables of participant age and batch and found that each of these features was well captured in the largest SVs (S1 and S2 Figs).

**Table 1:** Characteristics of relapsing-remitting MS cases and controls, and count of CD4+ and CD8+ T cell samples included in analyses.

	Cases	Controls
**Norway**		
N	46	46
Age ± SD	38 ± 9	37 ± 9
Female	46 (100%)	46 (100%)
Treatment naïve^a^	44 (96%)	-
On treatment	2 (4%)	-
CD4+ T cell samples available for analysis	46 (100%)	41 (89%)
CD8+ T cell samples available for analysis	46 (100%)	46 (100%)
**Australia**		
N	48	53
Age ± SD	40 ± 11	45 ± 16
Female	48 (100%)	53 (100%)
Treatment naïve	16 (33%)	-
> 3 months off treatment	12 (25%)	-
On treatment^b^	22 (42%)	-
CD4+ T cell samples available for analysis	48 (100%)	53 (100%)
CD8+ T cell samples available for analysis	22 (46%)	11 (21%)

^a^Three individuals in this group were previously on treatment (5, 4, and 2.5 years before inclusion).

^b^One individual in this group had unknown treatment status.

**Table 2:** Overview of number of samples used in each of the five analyses, with quality control (QC) and analysis metrics.

T Cell Type	Cases, N	Controls, N	Probes Passing QC	SVs	λ	Candidate Regions	M-value Cutoff
**All cases, regardless of treatment**							
CD4+	94	94	423,500	13	1.11	3,989	0.154
CD8+	68	57	415,676	10	0.95	3,564	0.217
**Treatment-naïve or off treatment for at least 3 months**							
CD4+	72	94	423,500	12	1.10	3,902	0.170
**Treatment-naïve**							
CD4+	60	94	423,500	11	1.18	4,271	0.178
CD8+	44	46	409,357	7	1.02	3,703	0.198

Columns indicate the number of case and control samples included in each analysis, the count of CpG probes passing QC filters, the number of estimated surrogate variables (SVs), genomic inflation factor λ with SVs as covariates, the number of candidate differentially methylated regions tested, and the M-value cutoff determined by the *Bumphunter* R package.

### DMP analysis confirms hypermethylation in CD8^+^ T cells for MS patients

**Fig 1:**
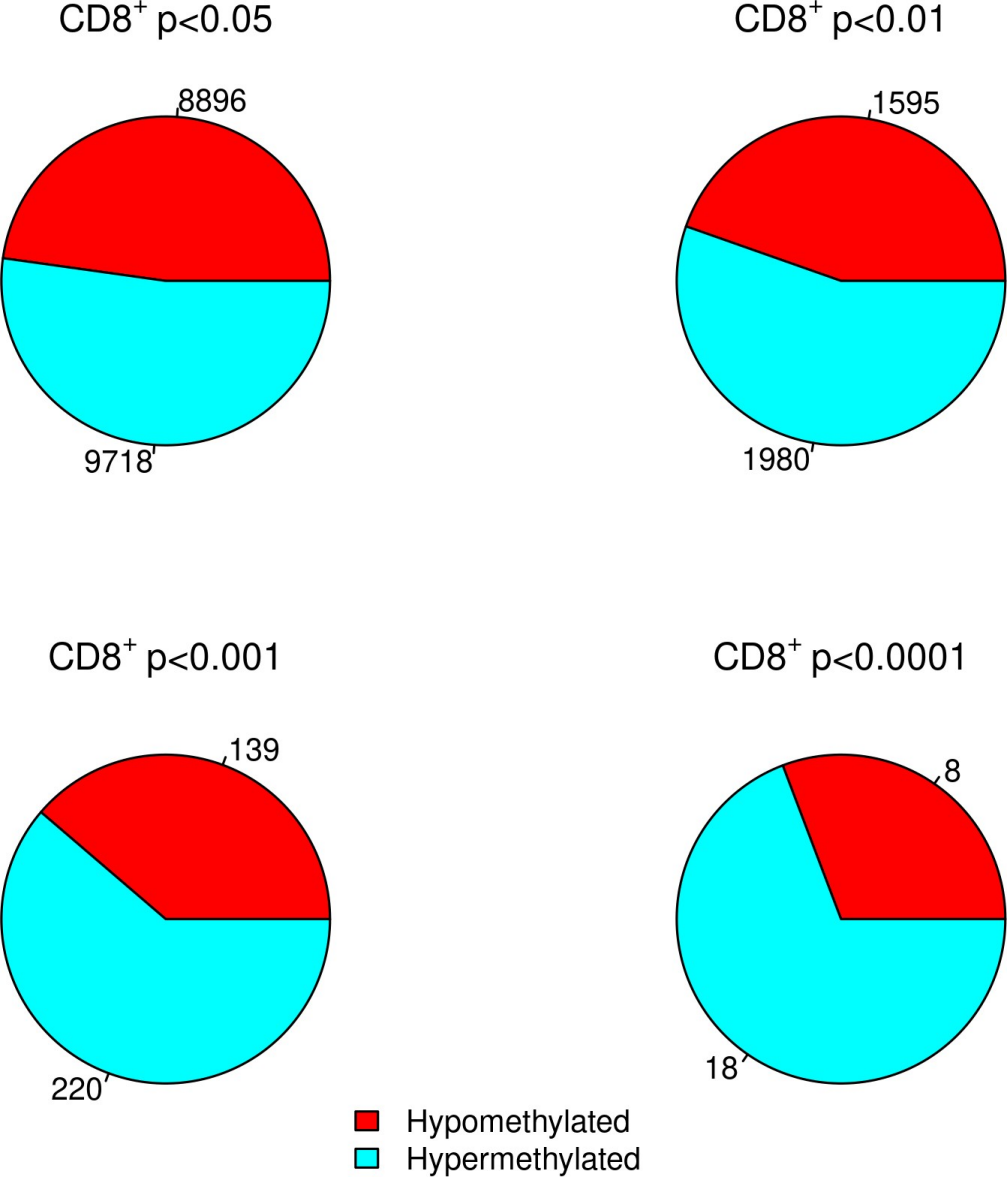
Proportion of significantly differentially methylated positions at increasingly stringent p-value cutoffs in the CD8^+^ T cells of 94 MS cases and 94 healthy controls. Numbers indicate the number of CpGs meeting the p-value threshold for hypomethylated and hypermethylated.

No individual DMPs were significantly associated with MS after adjusting for multiple hypothesis testing. However, when we focused on probes that showed a nominally significant p-value in the DMP analysis of all samples, we confirmed our previous findings[16] that CD8^+^ T cells of MS patients display a higher degree of DNA methylation as compared to healthy controls (Fig 1). This trend becomes increasingly apparent as p-values become increasingly stringent, ranging from 52% of sites hypermethylated at p<0.05 to 69% hypermethylated at p<0.0001. In CD4^+^ T cells no trend towards DNA hypermethylation was observed for any p-value cutoff.

### DMRs in MS patients compared to controls

As groups of CpG sites located near one another can be methylated or demethylated together, and identifying these regions of differential methylation is statistically more powerful than identifying single DMPs, we next sought to identify DMRs.[21] Results are summarized in Table 3. The exact same DMRs were identified for CD4^+^ T cells of cases not on treatment at the time of inclusion and CD4^+^ T cells of treatment-naïve cases (datasets c and d listed above), so only results for the latter are included here. Additionally, because microarray probes used to assess DNA methylation may be sensitive to SNPs in the probe sequences, we evaluated whether methylation at individual CpGs within DMRs corresponded to differences in genotypes. Out of 34 CpGs in DMRs with SNPs in the probe sequences that were also present in the imputed Norwegian genetic data, 4 CpGs in the *MOG*/*ZFP57* DMR were found to be differentially methylated by genotype. Dropping the 4 CpG sites resulted in a slightly higher family-wise error rate (FWER) for this DMR, but the result remained significant.

**Table 3:** Differentially methylated regions (DMRs) in CD4^+^ and CD8^+^ T cells between MS cases and controls.

DMR Genomic Position (hg19)	DMR Position Relative to Genes	# CpGs in DMR	Direction of Methylation Change in Cases	Patient Subsets with DMR in CD4+ Cells	Patient Subsets with DMR in CD8+ Cells
chr6:29648225–29649084	8kb downstream of *MOG*; 3kb upstream of *ZFP57*	18–22	Decreased	All patients	-
				Treatment-naïve only	-
chr6:32551749–32552453	Exon 2 of *HLA-DRB1*	7–8	Decreased	All patients	All patients
				-	-
chr12:739980–740338	Intron of *NINJ2;* first exon of *LOC100049716*	3	Increased	-	-
				Treatment-naïve only	-
chr17:33734664–33734664	3kb downstream of *SLFN12*	1	Decreased	-	-
				Treatment-naïve only	-
chr17:33759512–33760527	First exon *SLFN12*	11–12	Increased	All patients	All patients
				Treatment-naïve only	Treatment-naïve only

P-values were adjusted for multiple tests, controlling the family-wise error rate (FWER from *Bumphunter*), and a DMR was called if the FWER was less than 0.2. Dash (-) indicates an FWER>0.2.

#### Hypermethylation of *SLFN12* is associated with MS

**Fig 2:**
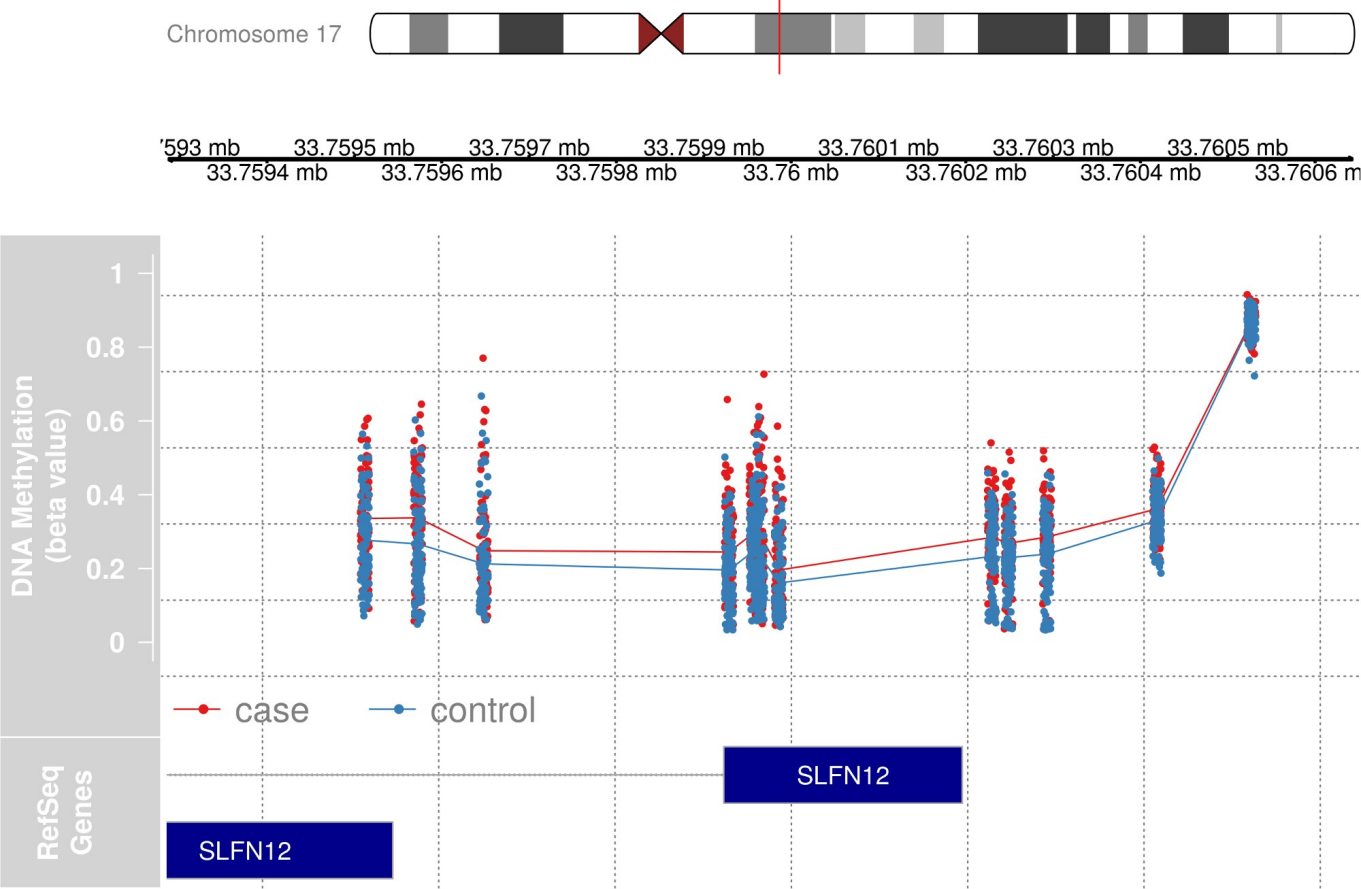
Detailed view of the differentially methylated region on chromosome 17, overlapping the first exon of *SLFN12*. Individual CpG sites and sample values from CD4^+^ cells from 94 cases and 94 healthy controls are represented by dots (red dots–cases, blue dots–controls), whereas the lines represent the average values on each CpG site. The position of two *SLFN12* gene transcripts are shown in dark blue. *Illustration*: *the gviz package for R*.

A consistent DMR signal was observed on chromosome 17 in CD4^+^ and CD8^+^ T cells (Table 3). A long DMR (between 18 and 22 differentially methylated CpGs, depending on the dataset analyzed) covering the first exon of *SLFN12* showed hypermethylation in MS patients compared to healthy controls in both CD4^+^ and CD8^+^ T cells (Fig 2). Hypermethylation was seen in all strata, regardless of treatment status of cases. A much smaller DMR consisting of a single CpG site hypomethylated only in the CD4^+^ T cells of treatment-naïve cases compared to controls was identified 3kb downstream of *SLFN12* (Table 3). We note that a DMR can consist of a single CpG site due to the width of the *Bumphunter* smoothing function.

#### Hypomethylation in the MHC region

Evidence for a DMR was observed in a regulatory region just outside the HLA Class I region on chromosome 6 in CD4^+^ T cells (Table 3). Specifically, this DMR is located in a regulatory region 8kb downstream of *MOG*, encoding myelin oligodendrocyte glycoprotein, which is expressed in myelin sheaths,[22] and 3kb upstream of the zinc finger protein gene *ZFP57*, encoding a protein that likely acts a as a transcriptional repressor (RefSeq, Sep 2009). In addition, we confirmed evidence of hypomethylation in the *HLA-DRB1* gene in MS CD4^+^ T cells compared to healthy controls, as previously reported,[20] as well as in CD8^+^ T cells (Table 3). However, the *HLA-DRB1* result was not observed when analyses were restricted to treatment-naïve cases.

#### Hypermethylation of the *NINJ2/LOC100049716* locus

A DMR consisting of 3 CpGs in the first intron of the *NINJ2* gene demonstrated hypermethylation in CD4^+^ T cells from treatment-naïve MS patients when compared to healthy controls. This region is overlapped by the first exon of an uncharacterized long non-coding RNA (*LOC100049716*).

### DMRs correspond to differential expression of genes in whole blood

**Fig 3:**
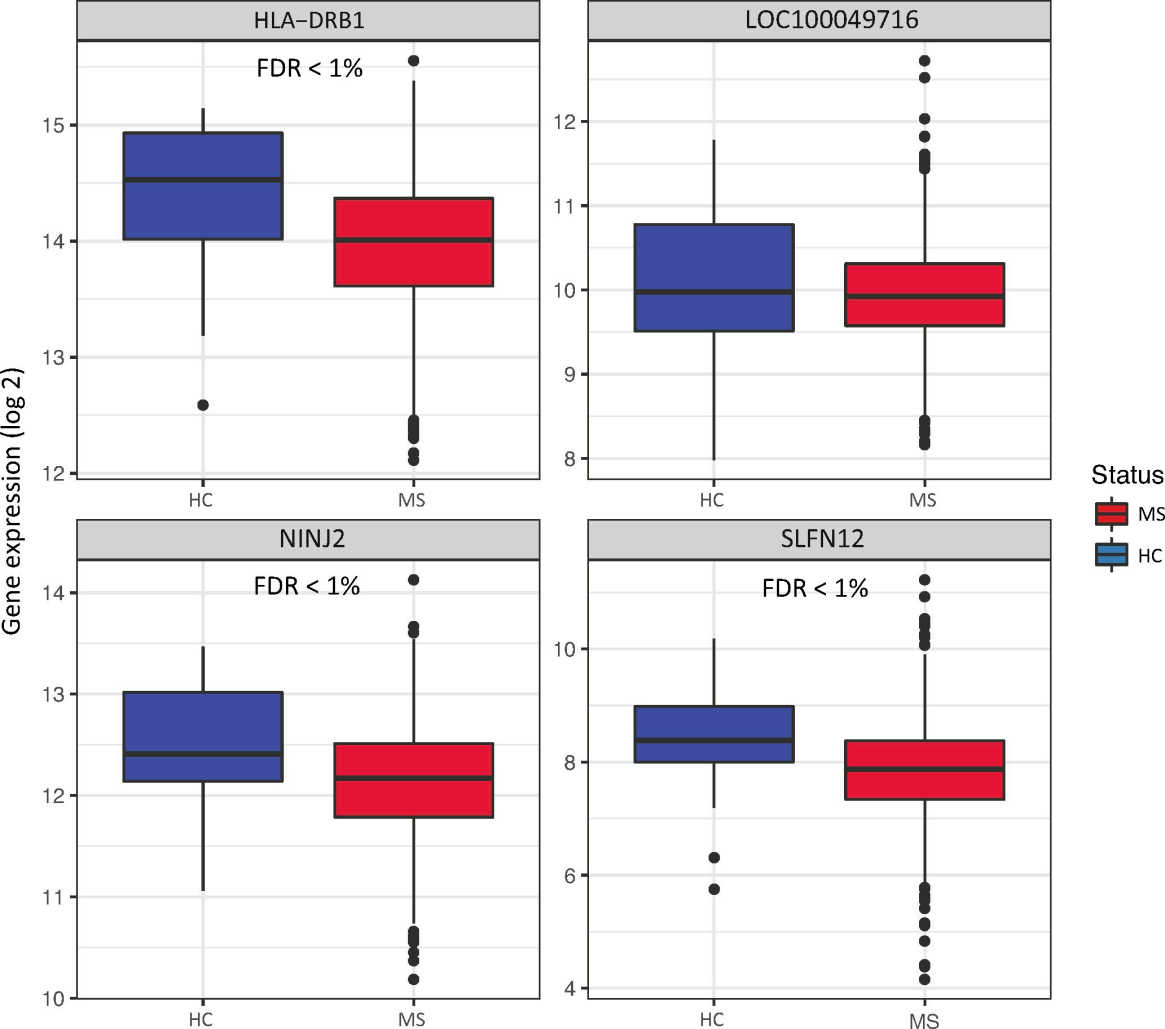
Gene expression levels of *HLA-DRB1*, *LOC100049716*, *NINJ2*, and *SLFN12* in whole blood of MS cases compared to healthy controls (HC). Horizontal lines of boxplots indicate the lower quartile, median, and upper quartile of log2 gene expression intensities; whiskers indicate the lowest and highest values within 1.5 times the inter-quartile range of the lower and upper quartiles; dots indicate outliers.

To assess whether the observed DNA methylation was associated with gene expression of nearby genes, we nominated six candidates in close proximity to the DMRs identified in the current study: *SLFN12*, *NINJ2*, *MOG*, *HLA-DRB1*, *ZFP57*, and *LOC100049716*. Using whole blood samples from a large collection of MS patients and healthy controls, differential gene expression was assessed for four of the genes. *MOG* and *ZFP57* expression levels were below the microarray background threshold (average log2 expression <4) and therefore not considered in our analyses. Lower gene expression was observed for *SLFN12*, *HLA-DRB1* and *NINJ2* in MS patients compared to healthy controls, and there was no difference in *LOC100049716* (Fig 3). Results from genome-wide analysis (*limma*) and individual linear regression fits are listed in Table 4.

**Table 4:** Summary statistics for case/control expression differences in whole blood for genes identified in DNA methylation analyses.

Gene	FDR (*limma*)	p-value (*limma*)	p-value (linear fit)	Moderated log fold change (*limma*)	Log fold change (linear fit)
*SLFN12*	3.29e-09	2.25e-10	4.88e-11	-0.417	-0.599
*HLA-DRB1*	7.98e-12	3.17e-13	1.28e-13	-0.302	-0.442
*NINJ2*	1.97e-05	3.64e-06	1.83e-09	-0.186	-0.349
*LOC100049716*	0.527	0.446	0.046	-0.038	-0.123

Genome-wide analysis (*limma*) and individual linear fit coefficients and p-values are listed, as well as *limma* p-values adjusted for multiple hypothesis testing to control the false discovery rate (FDR). *Limma* moderates fold changes and hence represent more conservative coefficients and p-values than linear fit.

## Discussion

Our findings show that CD4^+^ and CD8^+^ T cells isolated from MS patients have regions of markedly increased or decreased methylation compared to cells isolated from healthy controls. These regions may influence disease etiology and shed light on risk factors for MS.[3] Compellingly, a DMR flanking the first exon of *SLFN12* occurred in all patient subsets for both CD4^+^ and CD8^+^ T cells. *SLFN12* encodes a member of the Schlafen protein family, which is a family of proteins encoded by a cluster of five genes on chromosome 17. Type I IFNs induce the expression of Schlafen genes.[23] *SLFN12* has been shown to be downregulated during T-cell activation in primary human cells.[24] From clinical observations and genetic studies,[4,6,25–27] there is convincing evidence that MS pathology is driven by T-cells, and IFN beta type I is an approved therapy for MS, making *SLFN12* a biologically plausible gene of interest for MS. Experimental evidence shows that *Slfn8* knockout mice have lower expression of pro-inflammatory cytokines and are resistant to induced experimental autoimmune encephalomyelitis (EAE), the mouse model of MS.[28] Though *Slfn8* is a different member of the Schlafen family, its clear role in EAE makes *SLFN12* an appealing target for further research in MS.

**Fig 4:**
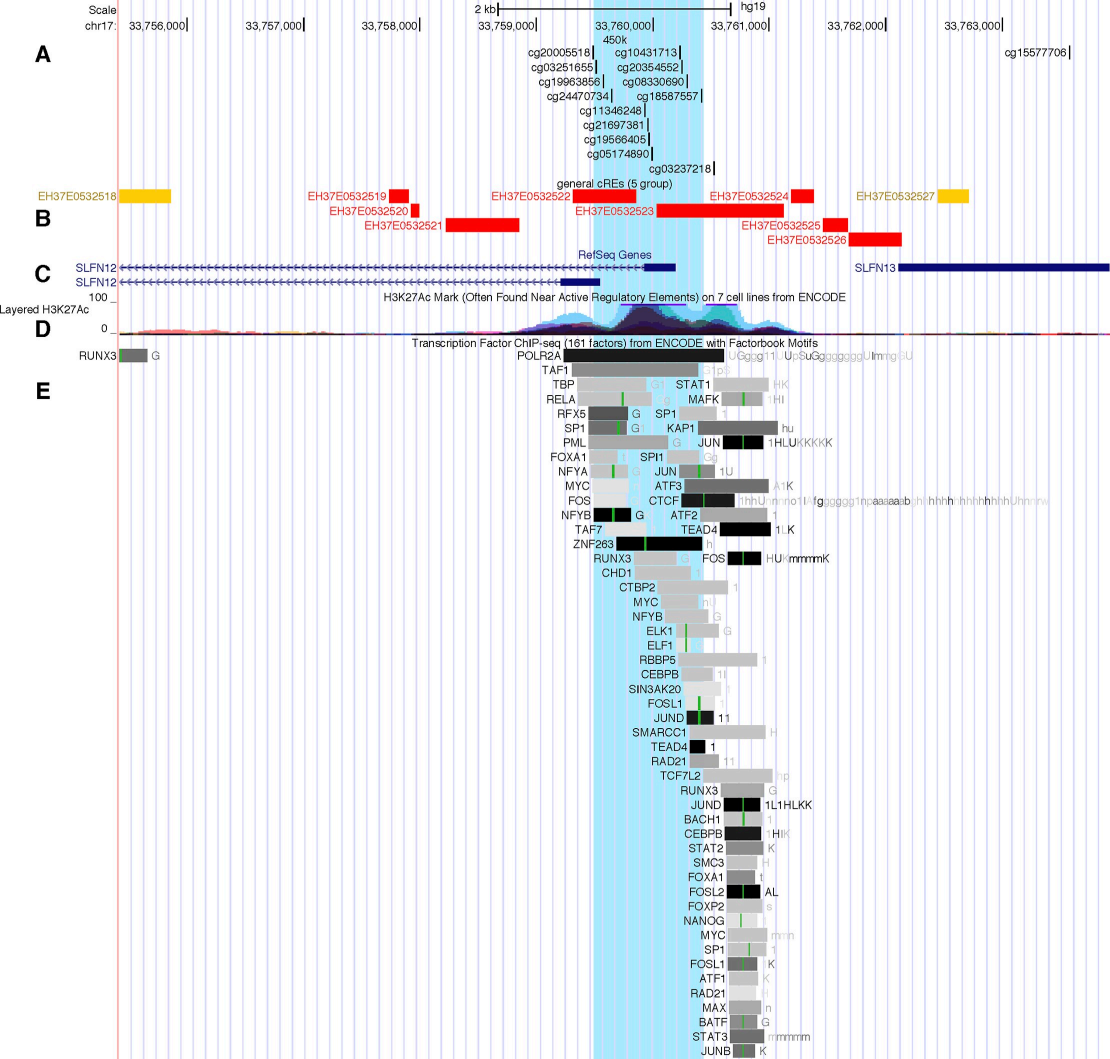
UCSC Genome Browser image showing the DMR in the *SLFN12* gene region highlighted in blue. Several tracks are shown: (A) the CpG sites on the Illumina 450k chip, (B) candidate regulatory elements identified by the ENCODE SCREEN algorithm, (C) the position of the *SLFN12* gene (the first exon of two transcript variants are shown), (D) the presence of the H3K27Ac mark across seven cell lines, and (E) the location of several transcription factor binding motifs in the genome sequence.

Hypermethylation of the first exon of *SLFN12* suggests repression of this gene in samples from MS patients, which is corroborated by decreased expression in whole blood of MS patients compared to controls (Table 4) in an independent cohort. It is not known whether this decrease in gene expression is caused by the observed hypermethylation or is a result of increased T-cell activity. A study of the transcriptional co-regulator gene *Mastermind-Like 1 (MAML1)* showed that its overexpression in embryonic kidney cells induced widespread methylation changes, including hypermethylation of *SLFN12* and corresponding downregulation of the gene, suggesting that a change in methylation alone could be responsible for decreased expression in T cells.[29] Conversely, a study in allergic rhinitis sufferers also found increased methylation and decreased expression of *SLFN12* in lymphocyte-enriched blood after participants were exposed to allergens, suggesting that T-cell activation could be the primary instigating factor.[30] Additionally, the area of the *SLFN12* DMR is enriched in the H3K27Ac histone mark, which is typically associated with regulatory elements, and it contains over 50 transcription factor binding motifs (Fig 4); increased methylation would therefore be expected to result in decreased expression.[31,32]

In line with earlier findings by Graves *et al*.[13] and Maltby *et al*.,[20] we confirmed that *HLA-DRB1* is hypomethylated in the CD4^+^ T cells of MS patients and observed the DMR for the first time in CD8^+^ T cells. *HLA-DRB1* is highly polymorphic, and is the strongest genetic risk for MS, raising the question of whether the observed differential methylation could be attributable to genetic variation in probe sequences used on the Illumina array. However, no CpGs with SNPs in probe sequences with differential methylation by genotype in this DMR were found. Interestingly, the *HLA-DRB1* DMR was identified using a different method from that described by Maltby *et al*. When we investigated the gene expression of *HLA-DRB1*, we observed that while hypomethylation was present in MS patients compared to controls, this gene has decreased expression in MS patients. This finding could be due to the location of the DMR in the gene body rather than a promoter,[33] or due to the fact that gene expression was investigated in whole blood rather than isolated T cells. Of note, the DMR was only detected when including all MS patients in the analysis regardless of treatment and not when restricting to off-treatment patients. This finding could be explained by lower statistical power in the off-treatment subgroups, or the DMR could result from medications used to treat MS.

Finally, hypermethylation of a region near *NINJ2* was observed for CD4^+^ T cells, which corresponded with lower expression of this gene in whole blood. This gene has previously been reported to show aberrant methylation in borderline personality disorder.[34] The DMR was only evident when off-treatment cases were included and was not detected when on-treatment cases were added, suggesting that treatment could be altering methylation in this region. This finding needs to be validated in an independent dataset.

When we compared the DMRs against the recently published 200 MS-associated SNPs[6] we did not observe any overlap outside of *HLA-DRB1*. It is possible that DNA methylation represents an independent functional mechanism of MS etiology. Though not in the list of 200 definite MS-associated SNPs, variants in *NINJ2* have been identified as “suggestive” MS-associated SNPs.[6] The increased methylation in this gene in CD4^+^ T cells from MS cases could be due to *NINJ2* variants, to an environmental factor, or to both, possibly acting in concert within an individual.

Some key strengths of this study were that searching for regions of differential methylation instead of isolated CpG sites provided greater statistical power, and that the relationships between our top DMRs and potential impact on mRNA levels were investigated a large independent dataset. In addition, the use of careful quality control procedures ensured that results were not due to technical artifacts. The use of SVA to infer covariates in the data allowed us to adjust for both measured and unmeasured confounders, and we confirmed that known variables such as measurement batch and BeadChip type were captured by SVs. Separate analysis of treatment-naïve cases allowed us to confirm that results were not due solely to use of medications. A limitation of this study was that DNA methylation was measured in cases after they developed MS, therefore temporality between methylation changes and disease onset could not be established. Also, environmental exposures were not evaluated. Methylation was assessed in T cells while expression was assessed in whole blood, which may not completely capture the relationship between DMRs and expression in T cells. Of the ~28 million CpG sites in the human genome, methylation was assessed only for sites on the 450k BeadChip.[35] Larger sample sizes will be needed to investigate differences in findings between CD4^+^ and CD8^+^ T cells. Finally, because this study was restricted to white females, findings may be sex specific and not generalizable to other populations.

Known environmental MS risk factors may exert effects on MS risk via changes in DNA methylation. For example, smoking increases MS risk, especially among carriers of HLA risk alleles or carriers of variants in *NAT1*.[3] Furthermore, smoking is associated with demethylation of the aryl hydrocarbon receptor repressor (*AHRR*) gene,[36] and the effect of smoking on demethylation of *AHRR* in blood is more pronounced in MS cases than healthy controls.[37] Larger effects from smoking on methylation throughout the genome have been observed in MS cases carrying HLA-DRB1*15:*01* and lacking the HLA-A*02 protective variant, and demethylation of a single CpG site near *SLFN12L* in former smokers relative to never smokers was detected in these cases.[37] Smoking is also associated with a more severe disease course.[3] These studies suggest that methylation could potentially be a mechanism by which smoking is acting to alter risk of disease or severity of disease course.

Other well-established MS environmental risk factors are associated with altered DNA methylation patterns and could help explain our findings. Adiposity has been found to be the cause of genome-wide methylation changes,[38] and adolescent obesity is associated with a two-fold risk of MS. Low vitamin D and decreased sun exposure are also associated with MS, and vitamin D can alter methylation status of other genes.[39] Several studies have shown an association of Epstein-Barr virus (EBV) with MS, and EBV exploits the epigenetic machinery of infected host cells to regulate its life cycle, resulting in widespread methylation changes to the host cell.[40] However, EBV resides in epithelial and B cells, so if methylation changes are due to EBV, they are more likely to be seen in those cell types. The impact of these environmental factors on methylation patterns specifically relevant to MS needs to be investigated further, in T cells and other immune cell types and tissues, as methylation changes may help explain the biological mechanisms through which environment affects disease risk, and consequently may identify new therapeutic targets that have not been revealed by genetic studies alone.

In conclusion, this is the largest genome-wide DNA methylation study of MS in CD4^+^ and CD8^+^ T cells to date. We show evidence that DNA methylation of CD4^+^ and CD8^+^ T cells plays a role in MS etiology. Consistent DMRs in *SLFN12* and *HLA-DRB1* were observed across two T cell sub-types, and differential gene expression was detected in whole blood for these gene candidates. Results indicate that DMRs may be detected in more accessible whole blood samples, paving the way for future large-scale studies of DNA methylation in MS. These findings would benefit from additional confirmation in larger independent case-control studies. Further research investigating the functional mechanisms underlying the association of these methylated regions to MS is warranted, particularly for *SLFN12*, which is not well-characterized.

## Methods

### Study populations

Norwegian patients with relapsing remitting MS diagnosed according to the McDonald criteria[41] (N = 46 females) were recruited from the Department of Neurology at Oslo University Hospital, Norway.[16] Controls (N = 46 females) were recruited through patients or from hospital employees and were frequency-matched to cases by age in 5-year increments. Almost all the Norwegian patients were treatment-naïve at the time of inclusion, except two patients were on IFN beta treatment, and three patients had previously received medications (none antibody-based, with a washout time of at least 2.5 years prior to inclusion).

Australian relapsing remitting MS patients were recruited from the John Hunter Hospital MS Clinic in New South Wales, Australia, and controls were recruited from the Australian Red Cross Blood Bank.[15,20] The Australian patients were a mix of patients who were treatment-naïve, off treatment for >3 months, or on treatment at the time of inclusion.

The Norwegian Regional Committee for Medical and Health Research Ethics and the Australian Hunter New England Health Research Ethics (05/04/13.09) and University of Newcastle Ethics (H-505-0607) committees approved this study. Methods were carried out in accordance with institutional guidelines on human subject experiments. Written and informed consent was obtained from all subjects.

### Purification of CD4^+^ and CD8^+^ T cells

For the Norwegian samples, CD4^+^ and CD8^+^ T cells were isolated from freshly collected peripheral blood mononuclear cells (PBMCs) using immunomagnetic cell separation selection kits (EasySep Human CD4^+^ T cell Isolation Kit (negative selection) and EasySep Human CD8^+^ Selection Kit (positive selection), StemCell Technologies, Canada) according to the manufacturer instructions. Purified cells were stained with FITC-conjugated mouse anti-human CD4 (clone RFT4, catalog #9522–02, Southern Biotech, USA) or FITC-conjugated mouse anti-human CD8 (clone HIT8a, catalog #555634, BD Biosciences, USA) and FITC-conjugated mouse IgG1 isotype control (clone 15H6, catalog #0102–02, Southern Biotech, USA) antibodies, and purity exceeding 95% was confirmed by flow cytometry (Attune Acoustic Focusing Cytometer, Applied Biosystems, USA).

For the Australian samples, CD4^+^ and CD8^+^ T cells were isolated from PBMCs using the same methods as the Norwegian samples. The purity of the cells was assessed by flow cytometry using a FITC conjugated anti-human CD4 antibody (clone OTK4, catalog #60016FI, StemCell Technologies, Canada) or an anti-human CD8 antibody (clone RPA-T8, catalog #60022FI.1, StemCell Technologies, Canada) on a BD FACSCanto II flow cytometer, then analyzed using FACSDiva software (BD Biosciences, USA) at the Analytical Biomolecular Research Facility of the University of Newcastle. All samples met a minimum purity threshold of >90%.

### DNA extraction

DNA from purified CD4^+^ and CD8^+^ T cell samples was extracted using QIAamp DNA Mini Kit (Qiagen, Germany) and bisulfite converted with the EZ DNA Methylation Kit (Zymo Research, USA). Methylation was assayed using Illumina BeadChips according to the manufacturer instructions (Illumina, USA). Two thirds of the Norwegian cohort were assayed with MethylationEPIC (EPIC) BeadChips. The Australian cohort and the rest of the Norwegian cohort were assayed with HumanMethylation450 (450k) BeadChips. Norwegian samples were assayed in four batches; Australian samples were assayed in two batches.

### Genotyping and imputation

The Norwegian samples were genotyped with the Human Omni Express BeadChip (Illumina). PLINKv1.09[42] was used to apply consecutive filters for per-SNP call rate (0.95) and per-sample call rate (0.95) prior to pre-phasing and imputation. MACH[43] was used for pre-phasing and genotypes were imputed against European samples from the 1000 Genomes data release 3 using Minimac3.[44] The Australian samples were not genotyped.

### DNA methylation data processing and analysis

The *Minfi* R package was used for pre-preprocessing, normalization, and quality control (QC).[45] EPIC and 450k datasets were combined by extracting the probes present on both platforms. The Norwegian and Australian samples were combined, and then datasets were split by cell type. Processing and analyses of CD4^+^ and CD8^+^ T cells were performed separately. Separate analyses were performed for a) treatment-naïve patients; b) those off treatment for >3 months at the time of inclusion in addition to treatment-naïve patients; and c) all patients. The *preprocessNoob* function was used for background subtraction and dye normalization, followed by quantile normalization with *preprocessQuantile*. Data points with detection p-value > 0.01 were replaced with “NA” values. CpG sites with more than 5% “NA” values across all samples were discarded. CpG sites with a common SNP (based on hg19, dbSNP build 144) at the CpG interrogation site or the single base extension were removed from analysis, as were sites with probes predicted to cross-hybridize to other genomic locations.[46] Predicted gender based on X and Y chromosome methylation matched each study participant’s gender. None of the samples had more than 5% “NA” values for sites forwarded for analysis.

Methylation beta values, defined as the proportion the methylated signal makes up of the methylated plus unmethylated signal, were logit transformed into M-values for analysis to reduce heteroskedasticity. Surrogate variable analysis (SVA) was run on each separate dataset analysis stratum to find latent variables. Such variables may represent batch effects, cellular heterogeneity or other unknown confounders (e.g., varying levels of inflammation). Measured potential covariates, such as age and batch, were not included when estimating SVs to allow SVA to identify such covariates directly from the data, as described by Jaffe *et al*.[47] and Leek,[48] with the “be” option used to determine the number of SVs to calculate, since this option resulted in lower genomic inflation compared to the “leek” option.[48] To determine DMPs, an epigenome-wide association analysis was performed using the empirical Bayes method in *limma*, with the M-value for each CpG as the outcome variable and disease status and SVs as predictors.[49]

Differentially methylated regions (DMRs) were identified using *Bumphunter*,[50] using the same outcome and predictor variables as in the DMP analysis. CpG sites separated by at most 500 bp were used to define clusters, and then 1,000 bootstrap samples were used to generate a null distribution of regions. Candidate regions were nominated with *pickCutoff*, using the 99% quantile of the null-distribution as a threshold. An adjusted p-value cutoff of 0.2 for the family-wise error rate (FWER) produced by *Bumphunter* was used to assign DMRs in each analysis. A liberal p-value cutoff was used because controlling the FWER (the probability of making at least one type I error) is more conservative than controlling the false discovery rate (which controls the proportion of type I errors). Imputed genome-wide SNP data was used to identify probes containing polymorphic SNPs in their recognition sequence for the Norwegian data. For each DMR, CpG sites with probes containing SNPs were further assessed for differential methylation by genotype. Those CpG sites were then excluded and supplementary DMR analyses were performed for the entire dataset.

### Whole blood gene expression data generation and pre-processing

Gene expression was evaluated in a separate population. Whole blood PAXgene tubes were collected from 1,329 female relapsing remitting MS patients at baseline of the phase 3 studies DEFINE and CONFIRM for demonstrating efficacy of delayed-release dimethyl fumarate for the treatment of RRMS, and from 97 female healthy volunteers.[51,52] These subjects were predominantly of European ancestry (87%) and treatment-naïve (77%). They had a median age of 39 years (inter-quantile range (IQR) 31–46) and median disease duration of 7 years (IQR 0–12). mRNA isolation, labeling and hybridization was done at Expression Analysis (Q^2^ Solutions, USA) in two batches. Labeled RNA was hybridized on Human Genome U133 Plus 2.0 Arrays (Affymetrix, USA). Sample data was processed using the R/Bioconductor *gcrma* library[53] and outliers were removed based on array quality scores. Expression data were normalized for technical factors—RNA quantity, quality (RIN score), and gene-level degradation slopes—and for batch effects, controlling for primary sample groups using the R *ComBat* library.[54]

### Differential Expression (DE) analysis

Probe-level expression data were summarized to gene-level data using the *collapseRows* function from the R/WGCNA library.[55] Gene-level DE analysis was performed using the R *limma* library.[49] Prior to the analysis, the data were adjusted for 48 surrogate variables[54] to adjust for latent variability in the data, controlling for disease status. For individual linear regression, the R function *lm* was used to regress gene expression levels of individual genes on disease status.

## Supporting information

S1 Fig Box plots of the first 6 surrogate variables (SV1-SV6) from the CD4+ T cell analysis of all participants according to batch. Batch is correlated with each of the first 6 SVs except SV5. Illumina chip type (450k vs. EPIC) appears to be captured particularly well by SV1.
(PDF)

S2 Fig Scatterplots of the first 6 surrogate variables (SV1-SV6) from the CD4+ T cell analysis of all participants according to participant age at the time of blood draw. Pearson correlation coefficients and p-values are given. SV3 and SV4 appear to capture age the best.
(PDF)
