# Supplementary material for: Increased DNA methylation of SLFN12 in CD4+ and CD8+ T cells from multiple sclerosis patients
Source: PLoS One. 2018 Oct 31;13(10):e0206511. doi: 10.1371/journal.pone.0206511 (PMC6209300; doi:10.1371/journal.pone.0206511)

S1 Fig. Box plots of the first 6 surrogate variables (SV1-SV6) from the CD4+ T cell analysis of all participants according to batch. Batch is correlated with each of the first 6 SVs except SV5. Illumina chip type (450k vs. EPIC) appears to be captured particularly well by SV1.

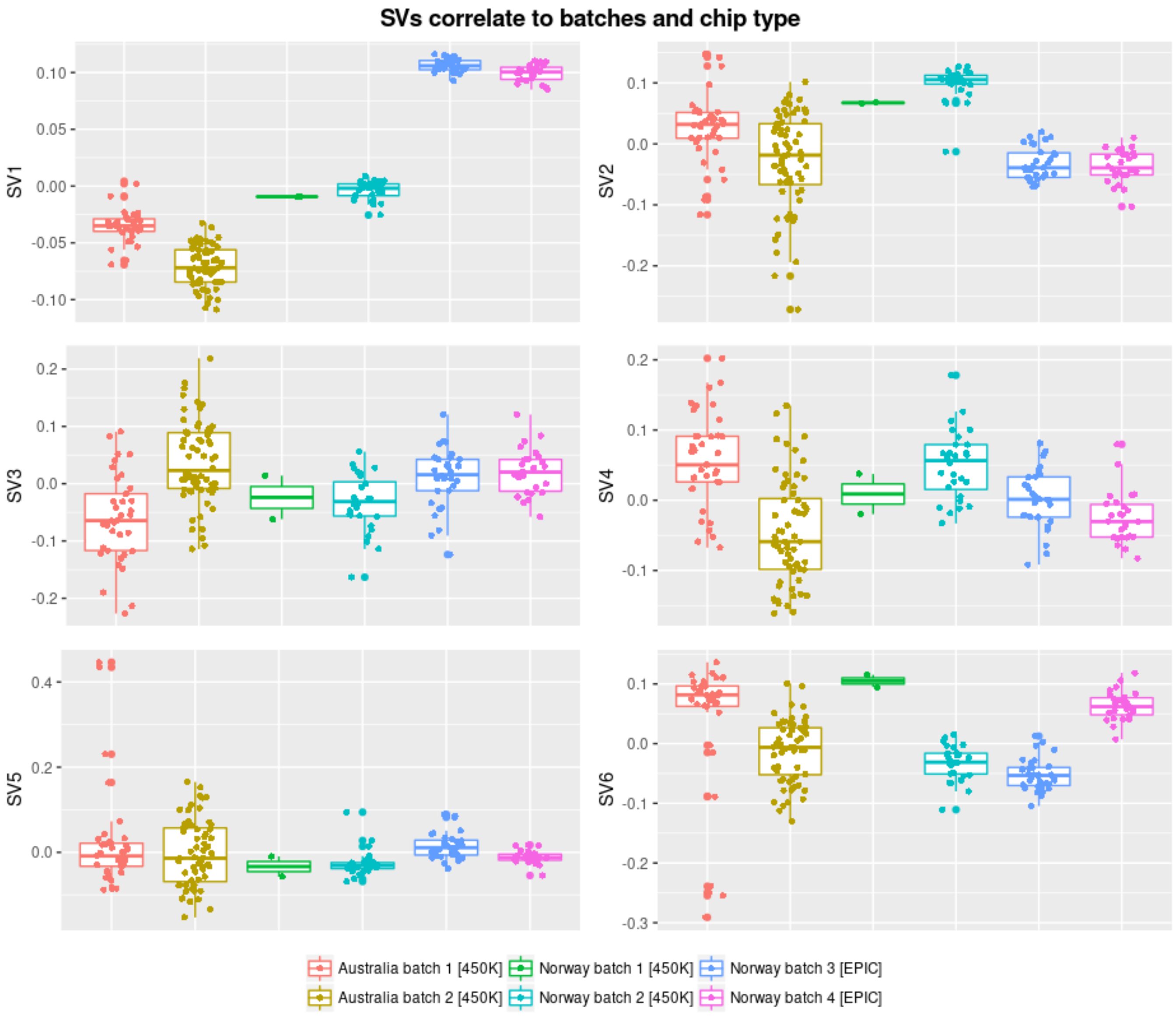

Supplement: S1 Fig — Batch is correlated with each of the first 6 SVs except SV5. Illumina chip type (450k vs. EPIC) appears to be captured particularly well by SV1. (PDF) [file pone.0206511.s001.pdf]
