# Supplementary material for: Increased DNA methylation of SLFN12 in CD4+ and CD8+ T cells from multiple sclerosis patients
Source: PLoS One. 2018 Oct 31;13(10):e0206511. doi: 10.1371/journal.pone.0206511 (PMC6209300; doi:10.1371/journal.pone.0206511)

S2 Fig. Scatter plots of the first 6 surrogate variables (SV1-SV6) from the CD4+ T cell analysis of all participants according to participant age at the time of blood draw. Pearson correlation coefficients and p-values are given. SV3 and SV4 appear to capture age the best.

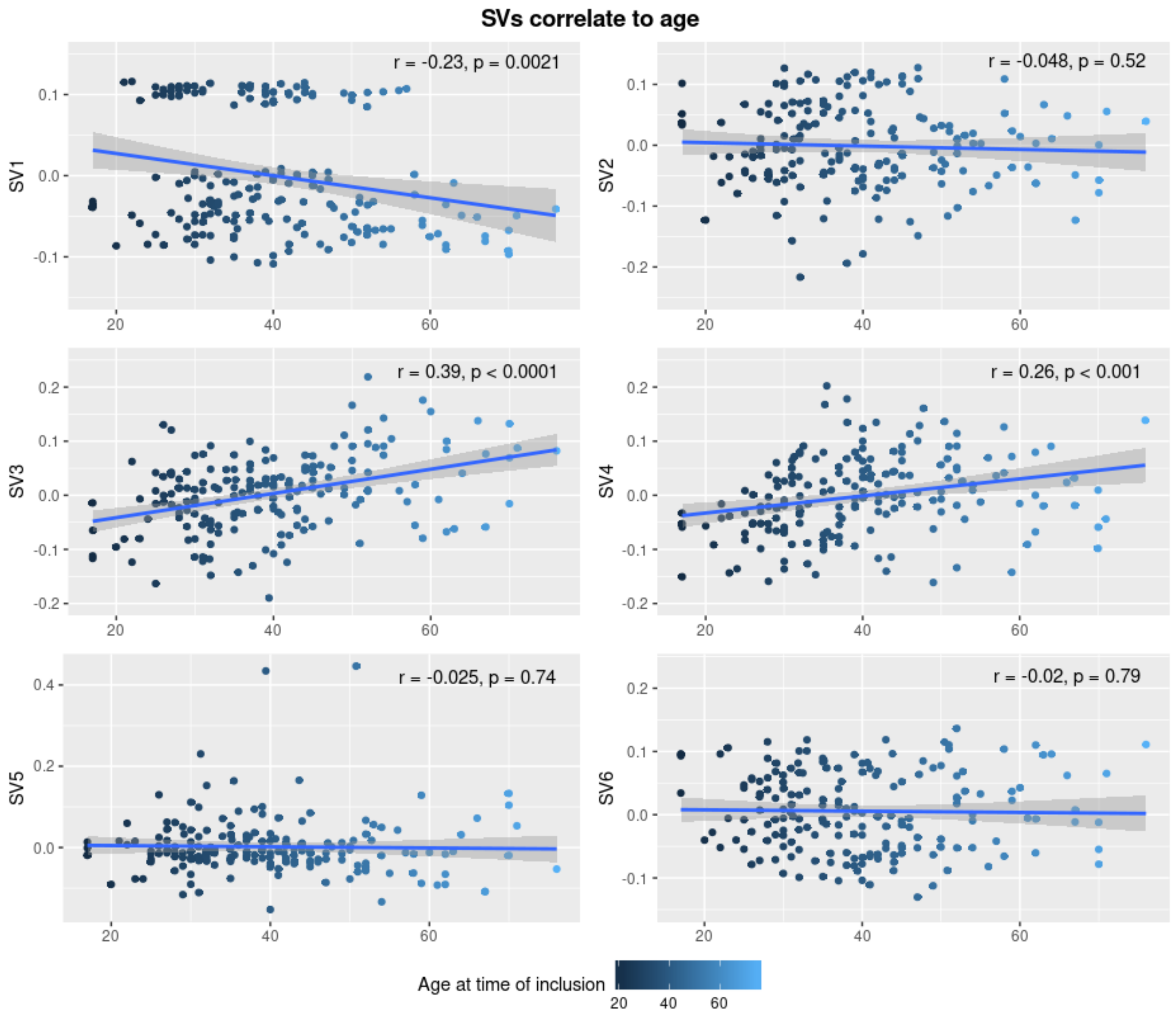

Supplement: S2 Fig — Pearson correlation coefficients and p-values are given. SV3 and SV4 appear to capture age the best. (PDF) [file pone.0206511.s002.pdf]
